# THE STRUCTURE OF CARE MANAGERS’ PRACTICE RESPECTING THE AUTONOMY OF THE FRAIL ELDERLY IN JAPAN

**DOI:** 10.1093/geroni/igz038.1865

**Published:** 2019-11-08

**Authors:** Chiemi Hata, Sachiko Kasahara

**Affiliations:** Shitennoji University, Habikino, Osaka, Japan

## Abstract

The objectives of current study are to clarify the structure of practice respecting the autonomy of the frail elderly under the Long-Term Care Insurance system in Japan and to discuss the related factors to the practice. The mailed self-administered questionnaire survey was conducted on 1398 care managers who working in In-Home Long-Term Care Support Providers in A City in Osaka with the condition that “office with multiple care managers engaged and one care manager with more than 5 years’ experience”. The response rate was 51.0% (713persons) and no missing data 615 (44.0%) was analyzed. Analysis was carried out using Mplus.ver8. The structure of practice respecting the autonomy of the frail elderly and the rerated factors were examined as a causal model using structural equation modeling. As the result, it was confirmed the goodness of fit to the data (RMSEA=0.049, CFI=0.927). By the confirmatory factor analysis, the care manager’s practice respecting the autonomy of the elderly was confirmed associating with three-factors structure; (1)data collection and assessment, (2) strength perspective and (3) professional relationship. Furthermore the practice was significantly affected by self-esteem of care-managers performance (β=0.494) and self-reflection to own work (β=0.269). In conclusion, the current study supported the hypothetical consideration in which self-esteem and self-reflection in care manager’s practice significantly affected the practice respecting the autonomy of the frail elderly.

